# Current Issues and Perspectives of Algae in Drinking Water Supply System: Colloidal Algae Is an Important Noticed Existence Form

**DOI:** 10.3390/microorganisms14051085

**Published:** 2026-05-11

**Authors:** Lijuan Wang, Shengnan Zhang, Yingying Han, Rixin Zhang, Weigao Zhao

**Affiliations:** 1 China Municipal Engineering North China Design & Research Institute Co., Ltd., Tianjin 300350, China; juan_1705@163.com; 2 School of Civil Engineering, Tianjin Ren’ai College, Tianjin 301636, China; shengnanzhang@tju.edu.cn; 3 The International Joint Institute of Tianjin University, Fuzhou, Tianjin University, Tianjin 300072, China; 2025439216@tju.edu.cn; 4 School of Environmental Science and Engineering, Tianjin University, Tianjin 300350, China; zrx1291_@tju.edu.cn; 5 State Key Laboratory of Pollution Control and Resource Reuse, School of the Environment, Nanjing University, Nanjing 210023, China

**Keywords:** algal blooms, colloidal algae, detection method, drinking water treatment plants, water sources

## Abstract

Algal blooms in water sources, exacerbated by global climate change and water eutrophication, pose a significant threat to water quality, ecological safety, and human health. Seasonal algae blooms in drinking water sources, particularly those in colloidal form, present substantial challenges to the safe and stable operation of drinking water treatment plants. To address these challenges and gain a better understanding, this study reviews current issues and perspectives on algae in the drinking water supply system (DWSS). Algal contaminations are more frequent and severe in the tropics and subtropics spatially, while temporally, they pose greater concern during summer and autumn. Moreover, various detection methods, including conventional and advanced techniques, are discussed based on the advantages and disadvantages in species identification, cell quantity, and morphology observation. Additionally, treatment processes in DWSS, particularly pre-oxidation and coagulation, are effective in removing most algae. Furthermore, judging from the characteristics, microalgae and Microcystis aeruginosa exist as colloidal algae among the whole process of DWSS, yet the physical states of algae have been largely overlooked in previous research. This study fills this gap by introducing colloidal algae as a distinct form and analyzing its detection and removal from a colloid science perspective. This review thus provides a new reference for targeted algae control in DWSS.

## 1. Introduction

Driven by global climate change and water body eutrophication, algal blooms in water sources have emerged as a persistent environmental concern [1]. Algae-contaminated water poses threats to human health and disrupts the operation of drinking water treatment plants (DWTPs) [2,3,4].

Different algal species lead to distinct challenges in drinking water supply systems (DWSS). Diatoms and filamentous cyanobacteria can obstruct filtration processes and impair coagulation-sedimentation. Certain cyanobacteria (e.g., *Microcystis*, *Aphanizomenon*) and various green algae generate unpleasant odors. Furthermore, cell lysis releases organic compounds that may form disinfection byproduct precursors. In addition to species identity, the physical states of algae also significantly affect DWSS. Algae primarily occur in floating, settled, and colloidal forms. Floating and settled algae are more readily removed, whereas colloidal algae—a relatively newly identified form—remain suspended owing to their small particle size, low specific gravity, and high stability [5]. These properties render them challenging to remove via conventional treatment processes and may pose greater risks to water quality [6,7].

To explore the research landscape and identify potential gaps, we conducted an exploratory literature search using CiteSpace (version 6.4.R1). The search was performed in the Web of Science Core Collection database, and the time range covered the recent five years from 2022 to 2026. The research terms were set as Topic Search = (“algae*”) and Topic Search = (“drinking water*”). Only journal articles and reviews in English were included. CiteSpace was used solely as an exploratory visualization tool to identify keyword co-occurrence and research trends, not for quantitative meta-analysis. The results showed that the first four keywords with the higher frequency of 453 obtained articles were “removal” (occurrence proportion: 18.3%), “*Microcystis aeruginosa*” (16.8%), “cyanobacteria” (9.7%), and “degradation” (8.6%), indicating that algal removal and cyanobacterial blooms are major research foci. In contrast, the same search using “colloidal algae” yielded almost no results, only one article in the past five years, and none directly addressing colloidal algae as a distinct form. No specific study directly addressed colloidal algae, even when the search was extended. Existing research has largely failed to differentiate algal physical states, treating colloidal algae as ordinary algae in detection, identification, and removal studies. However, based on our previous work on colloidal substances, algal physical states may influence their environmental behaviors. This aspect deserves further investigation.

To address this gap, this narrative review aims to: (i) clarify the contamination characteristics of algae in water sources, including their spatiotemporal distribution; (ii) summarize the detection, identification, and removal techniques universally used for algae, covering conventional and advanced detection methods as well as treatment processes described according to the order of water treatment; and (iii) discuss colloidal algae as a newly recognized existence state, explore potential targeted treatment techniques, and thereby enable efficient recognition of colloidal algae species and quantities, facilitate their purposeful removal, and contribute to prediction and early warning. Consequently, this review provides an important reference for proposing comprehensive prevention and control measures for algae in DWSS, with special attention to the colloidal form.

## 2. Profiles of Algal Contamination in DWSS

Algal contamination in water sources is a complex natural phenomenon influenced by multiple environmental factors [2,8]. Owing to different climates, algal species and quantities exhibit significant differences in various geographical areas, and algal blooms can occur under suitable external conditions [9,10]. Moreover, the differences in climate are mainly referred to the different temperature zones and seasons. Thus, this section analyzed the algal contamination from spatial and temporal categorization.

### 2.1. Profiles of Spatial Distribution

Natural conditions, particularly global climate change, critically influence algal growth [11]. Rising temperatures and CO_2_ levels directly promote algal proliferation, increasing the frequency, duration, and severity of blooms [12,13]; extreme weather events (e.g., thunderstorms, rainfall) further enhance nutrient transport into water bodies, exacerbating outbreaks [14]. Additionally, hydrodynamic conditions play an essential role [15]. For instance, stratification induced by dam operations or solar radiation suppresses vertical mixing and material exchange, creating a stable, warm, illuminated surface layer that favors the aggregation and proliferation of cyanobacteria [16].

Algal contamination varies significantly across climate zones (As shown in Table A1 and Table S1) [17,18,19,20,21,22,23]. Tropical regions experience the most severe and persistent blooms, and contamination is gradually expanding to higher latitudes with global warming [24,25]. Under different hydrological regimes, *Microcystis* prevails in wet seasons, while *Cylin-drospermopsis* occurs in relatively dry seasons [26]. In addition, *Microcystis*, *Raphidiopsis*, *Planktothrix*, *Dolichospermum*, *Pseudanabaena*, and *Aphanizomenon* were frequently occurring genera, which usually include multiple toxic species [27]. Specifically, *Raphidiopsis raciborskii* has increasingly been reported in Europe, North America, and South America throughout the year [28].

Temperate zones are characterized by distinct seasons compared with the tropical and frigid zones. Thus, there are more significant differences in the algal species in the four seasons, which are introduced in detail in the following Section 2.2. Especially, *Microcystis aeruginosa* spreads and dominates phytoplankton communities in temperate freshwater sources [29]. *Microcystis aeruginosa* shows a strong growth response to nutrient enrichment: while nitrogen or phosphorus addition individually stimulates proliferation, their combined addition synergistically maximizes biomass, underpinning its competitive advantage within phytoplankton communities [16].

Frigid regions are less favorable for algal growth and reproduction due to high lat-itude, low temperature, and insufficient light [30]. Consequently, reports on algal contamination in such persistently cold environments remain scarce. Nevertheless, some algae, such as *Chlamydomonas* sp. and certain diatoms, are adapted to local conditions [30].

### 2.2. Profiles of Temporal Distribution

Seasonal characteristics are evident in temperate regions, and algal growth exhibits marked seasonal variations (as shown in Table A1 and Table S2) [31,32,33,34,35,36,37]. Seasonal changes regulate the vertical position and growth characteristics of algae: they are generally benthic in winter and rise to accumulate at the surface in summer [38]. Low temperatures in early spring and late autumn are generally unsuitable for algal growth [39]. However, some algae thrive at low temperatures, such as cryptophytes, dinoflagellates, and diatom. Thus, these algae can also form blooms in spring or winter [40]. In contrast, late spring, summer, and early autumn—when water temperatures are higher—favor algal growth, making summer the most prone season for algal blooms in temperate zones.

Although algal contamination in spring gets less attention than that in summer, algal blooms are also present. This may be related to the fact that the growth of herbivorous zooplankton was inhibited in spring, thus, less algae were predated [39]. Besides, it is also associated with the morphological structure and physiological function of algae [40]. Specifically, algal blooms in spring are dominated by algae that can survive in low-temperature and low-light, such as dinoflagellates. Moreover, large-scale algal blooms dominated by *Microcystis* spp. occurred in Lake Taihu, which began in the late spring (April–May) and continued into the autumn (November) [41].

The most serious algal blooms tend to erupt in summer [42]. There are several promotion factors in summer. First, algae grow and reproduce faster at higher temperatures, so the frequency and persistence of algal blooms increase in summer [43]. Second, higher temperatures reduce the viscosity of the water, which produces higher floating speeds for algae [44]. Third, stronger and more persistent vertical stratification is beneficial to algal floating and growth. For example, since 2002, Lake Erie has experienced the most significant blooms in late summer almost every year [45].

Algal contamination in summer often continues into the autumn, but relatively few algal blooms begin in autumn. The most common and severe algal blooms in autumn are caused by cyanobacteria. Cyanobacterial outbreaks are not only related to relatively high temperature, but also related to the unique morphological structure and physiological functions of cyanobacteria [46]. Specifically, cyanobacteria have unique metabolic characteristics, fewer required nutrients, and stronger external contamination resistance [47,48]. Besides, cyanobacteria also exhibit advantages in cell structure and genome size, leading to greater growth advantages in algae community.

In winter, algal density and bloom areas are significantly lower than those in other seasons. Some algae continue to bloom in winter when the water temperature is low [49,50]. For example, Chlamydomonadales is a kind of green algae, which may grow in winter [51]. Moreover, due to the slower metabolism at low temperatures, the loss rate of algae reduces. Although algal cells remain inactive during winter, they will be able to regrow when the temperature rises. Thus, it is worth noting that algal contamination still should be controlled in winter [52]. It can not only reduce outbreaks of algal blooms in winter, but also prevent subsequent algal outbreaks in warmer seasons [53].

## 3. Detection and Identification of Algae in DWSS

**Figure 1:**
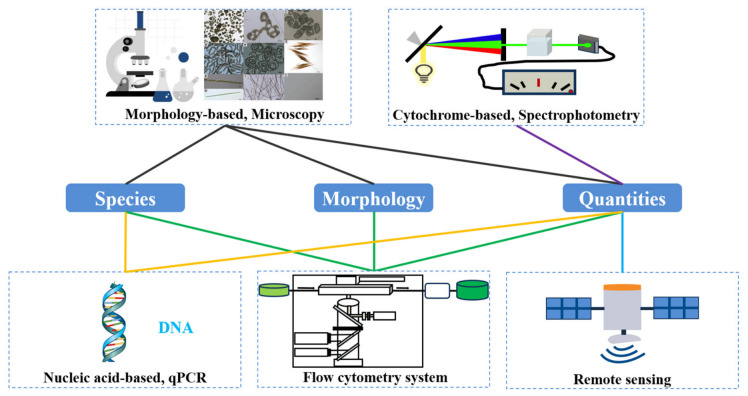
Comparisons of detection and identification techniques of algae in drinking water supply system. The microscopic figure of algae in the upper left was from Huo et al. [54].

To understand the existence of algae accurately and rapidly, so that appropriate warning and treatment measures can be taken, detection and identification of algae in DWSS is crucial. There are various methods for algal detection and identification based on morphology, cytochrome, nucleic acid, etc. (Figure 1) [54].

### 3.1. Morphology-Based Methods

Microscopy is the most frequently used method for algal detection. It allows direct visualization of cell morphology and, with fluorescent stains, can distinguish live from dead cells [55]. Meanwhile, algal sizes, growth state, surface morphology, and internal structure can be revealed. Microscopy offers the best taxonomic resolution, identifying algae to genus or species level [56]. However, it is time-consuming, requires trained personnel, cannot be performed in-situ, and depends on chemical stains [56,57,58]. Moreover, the morphology and structure of algae can easily change with cell growth and environmental variations, which leads to potential errors and reduced precision, especially for small-sized algae [58]. Atomic force microscopy (AFM) provides nanoscale 3D imaging and can probe surface properties such as stickiness, but it is more specialized and not suitable for routine DWTP monitoring [59,60]. Therefore, microscopy is indispensable for periodic verification in DWTPs but unsuitable for real-time early warning. Importantly, the sample preparation steps (centrifugation or filtration) may inadvertently remove colloidal algae.

### 3.2. Cytochrome-Based Methods

Spectrophotometry and HPLC measure chlorophyll or other pigments to estimate algal biomass [56,61]. Spectrophotometry has advantages of simplicity in operation, which is stable, accurate, swift, and cost-effective. However, the results sometimes deviate from the Lambert-Beer law, due to the impurities in water, and the change in the algal chlorophyll content in different growth stages. HPLC offers lower detection limits and high throughput, but provides no morphological or species information [56].

In practical applications, these methods have been used alongside other techniques for phytoplankton analysis. Moorhouse et al. studied phytoplankton community succession in the River Thames using spectrophotometry for chlorophyll-a concentration, while community composition was analyzed by HPLC, microscopy, and flow cytometry [62]. Takahashi developed an automated cell counter that measures chlorophyll autofluorescence for routine algal management [63].

Because extraction-based methods require laboratory work and are time-consuming, they are unsuitable for rapid in-situ detection. Therefore, real-time routine monitoring is necessary to obtain dynamic information and changes in algae [64]. In-situ fluorometers and online fluorescent probes address this limitation by enabling real-time monitoring with low error after calibration [65]. Choo et al. showed that calibrated fluorometer outputs correlate well with microscopic counts, making them suitable for detecting spatial and temporal changes in algal populations [28,65]. However, environmental and technical barriers can affect the reliability of the output.

### 3.3. Nucleic Acid-Based Methods

Nucleic acid-based methods, particularly qPCR, involve the extraction of DNA or RNA followed by amplification and comparison to reference databases for taxonomic identification [66]. The commonly used amplifiable gene fragments generally include 18S rRNA and 16S rRNA etc. In the end, taxonomic identification and data analyses are accomplished through comparison to the reference database [67]. Common molecular biology methods consist of polymerase chain reaction (PCR) [68] and quantitative polymerase chain reaction (qPCR) [69]. Fluorescence in-situ hybridization (FISH) [70], isothermal amplification (ITA) [71], gene chip [72], etc. are novel.

Molecular methods are particularly useful for morphologically similar or closely related species that are difficult to distinguish by microscopy. They are not constrained by morphology and can be used for relative quantification of large sample numbers [56]. Moreover, they are objective and efficient, and exhibit high sensitivity and accuracy. Thus, they are especially suitable for detecting multiple species or very low abundance algae [72]. More importantly, they can not only identify and characterize known species accurately, but also assist in the discovery of new species [57]. However, they require specialized equipment and relatively long processing times. Besides, they cannot distinguish live cells from dead or different growth states [28]. Moreover, the technology is still immature, such as the lack of unified standards, the differences in the selection of gene fragments, incomplete databases, etc. [67]. Furthermore, there are also several technical limitations, such as false positives, nonlinear relationship with measured DNA/RNA, contaminations, and inappropriate primer design [66].

### 3.4. Other Advanced Techniques

Conventional methods do not allow for morphological characterization and algal density detection simultaneously. Thus, emerging techniques provide more options with the advancements, such as remote sensing and flow cytometry systems. Besides, machine learning algorithms also provide a novel data analysis method to distinguish algae on the basis of existing data. Consequently, intelligence, automation, and versatility are future trends.

At present, there are various remote sensor carriers, such as ground-based, spaceborne, airborne, and unmanned aerial vehicle-based. Among them, satellite remote sensing enables the accomplishment of large-scale, long-term, and cyclical data collection. It can obtain information on chlorophyll concentrations, algal densities, bloom areas, and the spatial and temporal distribution of algae [73]. Moreover, remote sensing can be combined with image-processing algorithms based on artificial intelligence. Remote sensing captures the rapid changes of algal blooms in a short time [73], and has a broad range of applications for the prediction and warning of algal blooms [74]. Dev et al. developed a novel semi-analytical approach to estimate the concentration of cyanobacteria by remote sensing [75]. Particularly, remote sensing can be useful to cover large regions or inaccessible sites, e.g., private lands and remote sites [76]. However, the spatial resolution of data from spaceborne sensors is low due to the small surface area of most inland lakes and reservoirs, which may affect mapping accuracy. Consequently, the next goals for remote sensing are high efficiency, low power consumption, high resolution, and portability.

Traditional flow cytometry (FCM) can show the algal cell integrity, cell membrane permeability, or cell membrane damage. However, it also has limitations, such as cumbersome steps and low efficiency, and it cannot distinguish the three-dimensional characterization [58]. Flow cytometry system combines optics, fluidics, and electronic parts to automatically photograph, measure, and enumerate algal cells, which is convenient and intuitive [77]. E.g., Romero-Martínez et al. successfully detected planktonic algae in ship ballast water with a flow cytometer and microscope [78].

Machine learning can be used as a tool for further data analysis based on existing data. Many machine learning algorithms (support vector machines, random forests, neural networks, etc.) combined with image processing techniques have been widely applied to the recognition, classification, and prediction of algae [57]. These methods allow computers to automatically learn the characteristics of different algae based on existing algal images, then select and extract features [79]. Next, when it receives a new algal image, it can give classification results through computer image processing technology, which is simple and fast for later identification [57]. Furthermore, deep learning, represented by convolutional neural networks, has been particularly prominent in applications [80]. Lang et al. proposed a neural network-driven 3D detection approach that uniquely leverages holography to acquire 3D sampling data for algal identification, and proposed a novel DH-CNN architecture to enhance detection speed and precision [81]. Yadav et al. improved the traditional convolutional neural network. The dataset was expanded to 80,000 images, and the scheme could achieve 99.97% classification accuracy [82]. Consequently, machine learning is based on the previous abundant data, thus systematic biases can be avoided. However, since algal cell morphology varies in different algae growth stages, there are difficulties in creating the database based on the existing data [57].

### 3.5. Practical Considerations and a Tiered Framework for DWSS

The selection of a detection method must balance multiple practical dimensions: speed, cost, taxonomic resolution, and operational feasibility. The methods in supply Table S3 differ substantially in these respects. Based on this comparison and field evaluations reported in the literature, the following practical guidance can be offered for DWTP operators.

For real-time early warning of algal blooms, in-situ fluorometry is the most cost-effective and scalable option, despite its inability to identify species or assess viability [65,83]. For periodic verification of species composition and cell viability, microscopy remains irreplaceable, but its high labor demand limits sampling frequency [58]. For targeted surveillance of toxigenic cyanobacteria, qPCR provides high sensitivity and species specificity; however, its laboratory requirement and higher cost make it more suitable for confirmatory analysis than routine screening [84]. For source-water bloom mapping, remote sensing offers unique large-scale coverage, but it cannot detect subsurface algae and provides no species-level information [75,76].

Notably, different methods can lead to substantially different risk assessments. A multi-lake comparison showed that 78% of samples exceeded alert levels by fluorometry, compared to only 16% by ELISA for the same water samples [84]. This underscores that method selection directly influences management decisions. A tiered monitoring framework has been proposed to balance cost and information needs: low-cost probes for initial screening, microscopy and qPCR for confirmation, and toxin-specific assays only when health thresholds are exceeded [85].

## 4. Common Removal Techniques for Algae in DWSS

**Figure 2:**
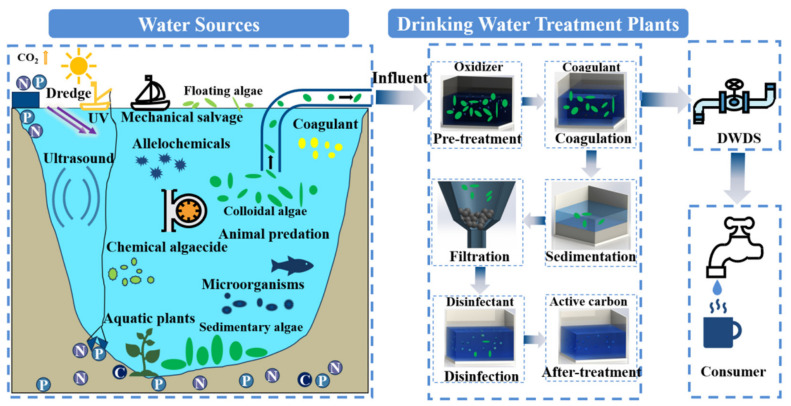
Common removal techniques for algae in DWSS. The C in the circle represents carbon, the N in the circle represents nitrogen, and the P in the circle represents phosphorous. The DWDS was the abbreviation of drinking water distribution system.

Algal growth and reproduction are unavoidable in natural water, thus, it is necessary to remove algae by comprehensive measures in DWSS to ensure the quality and safety of drinking water. Nowadays, algae removal methods are included at each stage of DWSSs. According to the order of water treatment, common removal technologies can be divided into treatments in water sources, pre-treatment processes, conventional treatment processes, and after-treatment processes in DWTPs, which are described in detail below (Figure 2).

### 4.1. Drinking Water Sources

There are various methods for removing algae in water sources, including physical methods, chemical methods, and biological methods (Table S4) [86,87,88,89,90,91,92,93]. Physical methods are simple but require intensive labor and cannot fully remove algae [94]. Xu et al. engineered a hybrid microfiltration enclosure system by integrating a mechanically-supported microfiltration membrane onto a physical containment structure. The composite membrane achieved a greater than 80% reduction in algal density within the permeate and significantly mitigated the risk of algal intrusion into downstream water treatment facilities [95]. Chemical methods are highly effective but risk secondary pollution and non-target toxicity [96]. Biological methods are eco-friendly and low-cost, yet suffer from slow response and variable efficacy. Coupling allelopathic substances with carrier materials to form sustained-release microspheres can ensure persistent and stable allelopathy, potentially serving as an effective strategy against future algal blooms outbreaks. Li et al. engineered a chitosan–gallic acid sustained-release algicide, which inhibited *Microcystis aeruginosa* by 99% and maintained control efficacy for 24 days [97].

### 4.2. Pre-Treatment Processes

Despite many measures taken at the water source, algae will inevitably enter the DWTPs. Thus, it is necessary to remove algal cells during the treatment process in DWTPs [4].

Pre-oxidation is commonly used to partially damage algal cells and improve subsequent coagulation efficiency. Typical pre-oxidants include permanganate, chlorine, and ozone at relatively low doses (e.g., 2.0 mg/L KMnO_4_, 1.0 mg/L NaClO or 0.5 mg/L Cl_2_) [98,99]. The goal is to reduce the negative surface charge of algae and promote floc formation, rather than complete cell inactivation. In addition to blooms removal, the above reagents are also used in preventing both *Microcystis aeruginosa* blooms in summer and *Cyclotella meneghiniana* blooms in autumn [100].

Dissolved Air Flotation (DAF) is a stable clarification method for algae with small particle size and low density [101]. Irem et al. functionalized DAF bubbles with amphiphilic polyoctyl chitosan (PO-chitosan), enabling high-efficiency, pH-independent removal of microalgae via enhanced bubble-cell interactions.

Other pre-treatment processes include ultrasonic and centrifugation. Peng et al. showed that ultrasound (740 kHz) disrupts Anabaena, improving coagulation while reducing disinfection by-products [102]. However, ultrasound is energy-intensive and noisy, and centrifugation risks cell lysis and toxin release [79].

### 4.3. Conventional Treatment Process

#### 4.3.1. Coagulation

Coagulation is one of the most researched processes with significant removal effects in DWTPs. A variety of coagulants are commonly used in DWTPs, including iron salts, aluminum salts, organic polymers, and biological coagulants [103]. Several factors influence coagulation efficiency, including coagulant properties [104], reaction conditions [105], and algal characteristics [106]. Some studies have shown that microplastics (MPs) can serve as nucleation sites, promoting the formation of larger coagulation flocs and neutralizing negatively charged pollutants. This mechanism significantly promoted algae coagulation and removal [107].

Traditional coagulation methods face challenges in removing algae effectively. For example, short hydraulic retention time, poor coagulant, and insufficient coagulant dosage all lead to insufficient capacity for coagulation. Thus, it is necessary to enhance coagulation. Main strategies include pre-oxidation, adding coagulant aids, improving coagulant types and dosages, optimizing hydraulic parameters and coagulation conditions, etc. [108]. Some studies show that the H_2_O_2_/Fe(II) oxidation-coagulation process, utilizing Fe(II) as a low-dosage coagulant, can efficiently remove microplastics and algae from water bodies [109]. Li et al. developed a red soil-based coagulant (RSC) via acid leaching and neutralization, achieving a 99.2% removal rate of *Microcystis aeruginosa* and outperforming conventional agents in treating eutrophic water bodies [110]. Lu et al. designed maleyl chitosan-graft-polyacrylamide (MHCS-g-PAM), and the optimal Chl-a removal rate was 98.6% at pH 7 [111]. Besides, magnetic flocculant is an emerging kind of flocculant, and under the action of the applied magnetic field, the aggregations settle and separate rapidly [103]. Bian et al. fabricated magnetic metal-organic frameworks (MMOFs) and polymeric ferric sulfate (PFS) (PFS-MMOFs) composites for the effective removal of *Microcystis aeruginosa*. The results showed that the coagulation effects are significantly improved under different hydrodynamic conditions [106]. More importantly, magnetic flocculation has been successfully applied in harvesting microalgae, thus it may achieve recycle and reuse of potential biofuel resources in DWTPs.

#### 4.3.2. Sedimentation

The sedimentation rate is largely related to the morphology and charge of algae [112]. Specifically, needle-shaped or filamentous algae have higher morphological resistance coefficients and relatively slow settling velocities. Thus, this morphological characteristic may increase the challenges in the removal process and reduce the treatment capacity of sedimentation. In addition, the surfaces of algae are mostly negatively charged, and the isoelectric points are proven to be around pH 3–4. Therefore, single algae are stable under the action of electrostatic repulsion, which is difficult to settle.

#### 4.3.3. Filtration

A better algae-water separation effect with less introduction of chemicals is the final goal, thus, filtration is of interest. There are many methods of enhanced filtration, such as changing the surface properties and size of algae, replacing filter media, optimizing filtration operating parameters, and adding filter aids [113]. Among them, filtering media is the most critical aspect in filtration. Moreover, cell retention and biodegradation in the filtering media are the most likely mechanisms for the removal of *Microcystis aeruginosa* and microcystin. Zhao et al. used glass beads as filtering media to remove *Microcystis aeruginosa*. It showed that there was no significant removal effect regardless of ionic strength, filter media size, and flow rate. Meanwhile, Zhao et al. found that there were risks of breakthroughs of algal cells, clogging of filters, and release of algal toxins [114]. Thus, filtration has limited effect on removing algae. It is necessary to search for environmentally friendly, efficient, easily accessible, and inexpensive alternative filter media, especially multi-layer and modified filter media.

#### 4.3.4. Disinfection

Disinfection is the final barrier for microbial safety in DWTPs, where any remaining algae must be completely inactivated. Chlorine is the most widely used disinfectant due to its low cost and persistent residual effect in the distribution network. However, excessive chlorine may cause algal cell lysis, toxin release, and formation of disinfection by-products [115]. Thus, it is necessary to select the appropriate type and dose of disinfectants [116]. The study demonstrates that the UV/PAA advanced oxidation process (AOP) effectively disinfects water, inactivates microalgae, and degrades algal toxins, offering a comprehensive solution for eutrophication and microbial contamination challenges.

### 4.4. After-Treatment Processes

The conventional treatment process has been applied widely, which dominates in removing algae. Moreover, there are also some discussions about algal removal in after-treatment processes, which mainly consist of advanced oxidation techniques and membrane technology.

Advanced oxidation processes (AOPs) are distinguished by the generation of highly reactive radicals that can degrade algal toxins and refractory organic matter. However, at present, it is difficult to be industrially applied owing to the low concentration of free radicals, long reaction time, huge material consumption, and high energy cost [105]. Therefore, the further application relies on the development of stable, low-cost, and mass-producible electrode material.

Membrane technology mainly consists of microfiltration, ultrafiltration, nanofiltration, reverse osmosis, etc. [59]. For instance, Zhang et al. implemented a two-stage ultrafiltration (UF) system downstream of a conventional sand filter, comprising a large-pore polysulfone hollow fiber membrane (200 kDa MW cut-off) and a fine-pore aromatic polyamide roll membrane (1 kDa MW cut-off), which effectively removed algae and algal organic matter under optimized conditions [117]. Generally, membrane processes do not destroy algal cells, so there is less release of algal toxins compared to chemical treatments. However, membrane contamination leads to a significant reduction in membrane permeability, thus, the membrane requires frequent cleaning and maintenance [105]. Moreover, waste streams from backwash and membrane cleaning may contain algal cells and algal toxins, which need to be treated carefully. Therefore, membrane filtration has been used relatively limitedly in DWTPs and is generally applied in household water purifiers.

### 4.5. Practical Considerations for Algae Removal in DWTPs

The comparison of the main removal technologies within DWTPs is provided in Table S5 [106,118,119,120].

The efficiency of each technology under real DWTP conditions varies considerably. Conventional coagulation-sedimentation is effective for many algae under optimal conditions, as summarized in recent reviews [105,121]. Conventional coagulation-sedimentation can achieve around 90% algae removal under certain conditions, while DAF can reach nearly 95% [121]. However, its efficiency drops sharply for small, negatively charged cells such as colloidal algae [122]. DAF performs well for low-density, buoyant algae, achieving high removal rates (>80%) in field studies [123], but its efficiency decreases for cells smaller than about 5 µm. Membrane filtration (UF/MF) can achieve >99% removal by size exclusion, but its performance is limited by membrane fouling, especially in raw water with high organic matter or turbidity [59,124].

Cost is another critical factor in technology selection. Coagulation with conventional Al/Fe salts is widely recognized as the lowest-cost option among mainstream treatment processes [85]. Pre-oxidation adds a marginal cost but can reduce coagulant demand, partially offsetting the expense [98,99,125]. Membrane filtration and advanced oxidation processes (AOPs) are significantly more expensive and are usually reserved for polishing or toxin degradation rather than bulk removal [124,126].

As for scalability (the ability to operate reliably at full scale), the technologies differ markedly. Conventional processes (coagulation, sedimentation, filtration, disinfection) are already operating at full DWTP scale worldwide. DAF has been implemented in large plants with stable performance [127], and enhanced coagulation (magnetic flocculation) has shown promise at pilot scale, though full-scale validation remains limited [128]. In contrast, ultrasonic and centrifugal methods remain at laboratory scale and are not feasible for routine DWTP operation [79,102]. Membrane filtration is scalable but requires careful fouling management and high capital investment, which currently limits its widespread adoption [124].

## 5. Colloidal Algae-Definition, Detection Challenges, and Treatment Implications

### 5.1. Definition

In the 19th century, Graham systematically investigated colloidal systems and first introduced the scientific concept of “colloid.” In the field of water environment research, colloids are typically defined as a special dispersion state with particle sizes ranging from 1 nm to 1 μm. Colloids exhibit several distinctive properties, including the Tyndall effect, electrophoretic mobility, and colloidal pump effect [129]. In aquatic environments, various pollutants can exist in a colloidal state under changing environmental conditions. Recognizing that a definition based solely on particle dimensions is often insufficient, our research group was the first to propose the concept of colloidal contaminants in drinking water systems, and has since defined colloidal microplastics and colloidal heavy metals according to their existence state [7,130,131]. Following this logic, the present review extends the concept to algae. Accordingly, colloidal algae are defined as algae that exhibit the aforementioned colloidal properties, namely stable suspension, reduced gravitational settling, and pronounced interfacial activity. It is acknowledged that there is currently no universally accepted operational definition for colloidal algae in terms of size range, stability criteria, or surface charge behavior. This knowledge gap should be addressed in future research.

Importantly, this colloidal state is not a fixed taxonomic attribute. The same species can exist as single colloidal cells under conditions of low ionic strength and stable shear, but form large colonies or aggregates in response to environmental changes such as variations in pH or cation concentration [132]. *Microcystis aeruginosa* cultured in the laboratory can remain stably suspended for long periods [114]. This stable suspension is attributed to a net negative surface charge. Gonçalves et al. measured the zeta potential of several microalgae and cyanobacteria, including *M. aeruginosa*, and reported a value of approximately −40.8 ± 4.4 mV for all studied suspensions, indicating a consistently negative surface charge [133].

To avoid confusion, colloidal algae should be distinguished from three other common forms in DWSS. Individual non-colloidal planktonic algae are generally larger and rely on flagella or gas vesicles for active movement or buoyancy, as seen in dinoflagellates and colonial *Microcystis* [134]. Algal aggregates or flocs, by contrast, form through bridging by extracellular polymeric substances or coagulants, typically exceed 50 μm, and settle rapidly [135]. A third category, algal-derived colloidal organic matter, consists of non-living macromolecules such as polysaccharides and proteins, which lack cellular structure and range from 1 nm to several hundred nanometers [136,137].

Direct monitoring studies on colloidal algae are still limited, making it difficult to discuss their spatial and temporal distributions. Nevertheless, indirect evidence supports their occurrence. For instance, when sampling water at a certain depth, the collected algae are mainly suspended colloidal algae due to their position in the water column [28].

### 5.2. Detection

Since colloidal algae are a newly recognized algal state, there are no targeted techniques for their detection and identification so far. However, based on the physicochemical characteristics that define the colloidal state, the applicability of existing methods can be rationally assessed. Optical microscopy and flow cytometry have been successfully applied to quantify suspended picophytoplankton and identified as useful methods for assessing phycocyanin-rich and phycoerythrin-rich picocyanobacteria in lakes [133,138]. In contrast, colloidal algae remain suspended in the water column rather than accumulating at the surface, so remote sensing—which relies on surface reflectance—is ineffective for detecting them [139]. Monitoring colloidal algae presents challenges beyond their biological activity, extending to how their unique physical structures respond to environmental changes and influence their behavior in water bodies.

Lesco et al. [140] employed advanced chromatography and field-flow fractionation to elucidate how salinity shifts trigger transitions of algal EPS between dispersed and aggregated states, thereby altering particle size and sedimentation rates. These insights could help optimize in-situ monitoring parameters. The optical measurability of colloidal algae remains underexplored. Studies on gelatinous colonies of brown cyst algae show that the optical activity of the colony wall derives from its constituent particles, while the internal fluid’s optical properties are influenced by dissolved colloidal organics [141]. This hierarchical relationship implies that monitoring should account for both the behavior of dispersed cells and their optical contributions as macroscopic building blocks, offering a potential pathway from colloidal-scale properties to remote sensing pixel scales, though validation for naturally occurring colloidal algae is still needed.

### 5.3. Treatment Implications

Since currently used algae removal methods are not species-specific, some of them, such as coagulation and membrane filtration, may in principle be applicable to colloidal algal removal due to their small size and surface charge, but direct experimental validation is lacking. Colloidal algae can be stably suspended due to electrostatic repulsive forces, making it more difficult to eliminate [7]. Consequently, conventional sedimentation processes are ineffective for their removal [142]. In contrast, magnetic flocculants have been shown to accelerate floc settling and offer a potential approach for colloidal algae removal [103]. Previous studies have also indicated that divalent cations such as Ca^2+^ and Mg^2+^ can promote algal aggregation through specific binding with extracellular polymeric substances (EPS), or via charge neutralization [143]. Therefore, under certain conditions, these ions may facilitate the aggregation of colloidal algae [144]. Besides, although the currently used filtration methods did not show significant algae cell removal [114], there was less addition of chemicals and less susceptibility to secondary contamination in filtration. Thus, it is worth exploring novel filtering media to remove colloidal algae in DWSS. In addition, based on the goal of being green and healthy, appropriate methods should be selected according to algal density. Specifically, intensive filtration without introduction of chemicals should be adopted at low density stage, which can be regarded as routine processing. Besides, intensive flocculation should be adopted at high density stage, which can be used as emergency processing.

## 6. Conclusions and Perspectives

Water quality issues induced by algal blooms have plagued DWSS for a long time, and posed potential hazards to residential water use. This review mainly investigated the status of algal blooms, as well as summarized the techniques of detection, identification, and removal of algae in DWSS. Moreover, colloidal algae represent a newly recognized physical state whose unique stability and adhesion could, in principle, influence their transport and removal in DWSS. However, direct evidence for their occurrence, behavior, and associated risks in full-scale DWTPs is currently very limited. It is noted that in DWSS, the physical state of pollutants has been largely overlooked relative to their chemical identity, even though this state critically determines contaminant behavior. Dedicated control strategies and engineering validation for the colloidal state are therefore needed. Therefore, the separate discussion of colloidal algae in this review is intended to highlight this knowledge gap and to call for targeted validation studies.

In summary, first, algal existence demonstrates significant spatiotemporal features due to climatic variations. For the spatial profiles, algal contaminations are more frequent and severe in the tropics and subtropics, and for the temporal profiles, they cause more concern in summer and autumn. Besides, microalgae in water sources and *Microcystis aeruginosa* in the laboratory exhibit the characteristics of colloidal algae. Second, the advantages and disadvantages, and applicable scenarios of five kinds of common detection methods are analyzed from species identification, cell quantity, and morphology observation. Among them, microscopy and qPCR are suitable for algal detection and identification in DWTPs. Moreover, remote sensing is not suitable for the detection of colloidal algae since colloidal algae usually suspend in natural water. Third, the removal effect of algae in each treatment process in DWSS was introduced sequentially. Among them, pre-oxidation and coagulation can remove most algae. In particular, magnetic flocculation has shown promise for the removal of colloidal algae under laboratory conditions, but full-scale validation is still needed.

To better ensure the safety of drinking water, it is important to detect algal species and quantities accurately and timely. Besides, daily monitoring for water quality, especially in-situ detection and online feedback, should be carried out in DWSS to provide references for algal timely assessment and blooms early warning. Although there are few specific studies on colloidal algae so far, according to the characteristics of colloidal substances, many algae may exist in the form of colloidal algae in DWSS. Moreover, by analogy with other colloidal substances, colloidal algae may pose higher risks, calling attention to colloidal algae in DWSS. Thus, future research should first develop and validate detection methods for colloidal algae, with standardization to follow. Besides, it is crucial to develop environmentally friendly, efficient, and economical removal techniques according to the species and quantities of colloidal algae. Moreover, the enhancement of the filtration process, combined with pre-oxidation and coagulation, holds great promise for improving the removal efficiency of colloidal algae. Furthermore, the removal rate of colloidal algae can be improved by intensive coagulation (high algal density, emergency process) and advanced filtration (low algal density, routine process) with less introduction of chemicals. Therefore, to better ensure drinking water safety, the algae problem in the whole process of DWSS, especially colloidal algae, should be targeted and comprehensively considered.

## Data Availability

The original contributions presented in the study are included in the article, further inquiries can be directed to the corresponding author.

## Supplementary Materials

The following supporting information can be downloaded at: https://www.mdpi.com/article/10.3390/microorganisms14051085/s1, Table S1. The reports of algal contamination in different temperature zones; Table S2. The reports of algal contamination in different seasons; Table S3. Comparison of algal detection methods for DWSS; Table S4. Common removal techniques for algae in drinking water sources; Table S5. Comparison of algae removal technologies within DWTPs.

## Abbreviations

The following abbreviations are used in this manuscript:

DWTPs	Drinking water treatment plants
DWSS	Drinking water supply system
DWDS	Drinking water distribution system
FCM	Traditional flow cytometry
HPLC	High-performance liquid chromatography
AFM	Atomic Force Microscopy
PCR	polymerase chain reaction
qPCR	quantitative polymerase chain reaction
DAF	Dissolved Air Flotation

## Appendix A

**Table A1:** Summary of spatiotemporal patterns of algal contamination in drinking water sources.

Dimension	Condition	Key Trends	Dominant Species	Reference
Spatial	Tropical subtropical	Highest frequency and severity, year-round occurrence	*Microcystis*, *Cylindrospermopsis*, *Raphidiosis*	[17,20,21,145]
	Temperate	Strong seasonality; blooms mainly in summer/autumn	*Microcystis aeruginosa* (summer); *diatoms*, *dinoflagellates* (spring)	[29,38,40]
	Frigid	Low algal activity; limited species	*Chlamydomonas* sp., *diatoms*	[30]
Temporal	Spring	Moderate blooms; cold-tolerant taxa dominate	*Diatoms*, *dinoflagellates*, *cryptophytes*	[39,40]
	Summer	Most severe blooms; cyanobacteria dominate	*Microcystis*, *cyanobacteria*	[43,44,45]
	Autumn	Blooms persist from summer, still significant	*Cyanobacteria*	[146]
	Winter	Low activity, but overwintering possible	Chlamydomonadales, *Aphanocapsa*, *diatoms*	[51,147,148]
