# Supplementary material for: Current Issues and Perspectives of Algae in Drinking Water Supply System: Colloidal Algae Is an Important Noticed Existence Form
Source: Microorganisms. 2026 May 11;14(5):1085. doi: 10.3390/microorganisms14051085 (PMC13210159; doi:10.3390/microorganisms14051085)
Supplement: Supplementary file 1 [file microorganisms-14-01085-s001.zip › microorganisms-4225872-supplementary.pdf]

Current issues and perspectives of algae in drinking water supply system: Colloidal algae is an important noticed existence form

Lijuan Wang [1,†], Shengnan Zhang [2,†], Yingying Han [3], Rixin Zhang [4] and Weigao Zhao [4,5,*]

[1] China Municipal Engineering North China Design & Research Institute Co., Ltd. Tianjin 300350, China; 724196591@qq.com

[2] School of Civil Engineering, Tianjin Ren'ai College, Tianjin 301636, China; shengnanzhang@tju.edu.cn

[3] The International Joint Institute of Tianjin University, Fuzhou, Tianjin University, Tianjin 300072, China; 2025439216@tju.edu.cn

[4] School of Environmental Science and Engineering, Tianjin University, Tianjin 300350, China; zrx1291_@tju.edu.cn, zhaoweigao@tju.edu.cn

[5] State Key Laboratory of Pollution Control and Resource Reuse, School of the Environment, Nanjing University, Nanjing 210023, China; zhaoweigao@tju.edu.cn

* Correspondence: zhaoweigao@tju.edu.cn

† These authors contribute equally to this work.

Content: Supplementary data includes 5 tables and reference in Page 2 – 15 :

Table S1. Page 2

Table S2. Page 3

Table S3. Page 4

Table S4. Page 6

Table S5. Page 7

References Page 9

Table S1. The reports of algal contamination in different temperature zones.

Temperature zone	Region	Country	Latitude	Dominant species	Reference
Tropical zone	Amba River	India	18°N	*Coscinodiscus oculus-iridis*	[1]
Tropical zone	Lake Naivasha	Kenya	0.7°S	*Cyanobacteria*	[2]
Tropical zone	Lake Victoria Nyanza	Kenya	1°S	*Microcystis*	[3]
Tropical zone	Pernambuco state	Brazil	7°-9°S	*Microcystis aeruginosa*	[4]
Subtropical zone	Lake Qarun	Egyptian	29°-29.5°	*Gymnodinium lantzschii, Prorocentrum aporum, Prorocentrum micans*	[5]
North temperate zone	Poyang Lake	China	28°-30°N	*Chlorophyta, Cyanobacteria, and Bacillariophyta*	[6]
North temperate zone	Lake Taihu	China	30°-32°N	*Microcystis*	[7]
North temperate zone	Pengxi Bay	China	31°-32°N	*cyanobacteria*	[8]
North temperate zone	Lake Songhua	China	42°-43°N	*Microcystis aeruginosa*	[9]
North temperate zone	Lake Erie	America, Canada	42°N	*Microcystis*	[10]
South temperate zone	122 water bodies	Argentina	24°-43°S	*Microcystis aeruginosa*	[11]
North frigid zone	Skagerrak	Arctic Ocean	-	*Diatoms*	[12]
South frigid zone	Lake Bonney	Antarctica	-	*Chlamydomonas sp.*	[13]

Table S2. The reports of algal contamination in different seasons.

Season	Water Resource	Country	Time	Dominant species	Reference
Spring	Xiangxi Bay	China	March - May	*Diatoms; dinoflagellates; green algae; cyanobacteria*	[14]
Spring	Lijiahe Reservoir	China	April	*Dinoflagellate*	[15]
Summer	Lake Nieuwe Meer	Netherlands	-	*Microcystis*	[16]
Summer	Lake Taihu	China	April - November	*Microcystis*	[17]
Summer	Lake Hulun	China	August	*Microcystis*	[18]
Summer	Lake Erie	America, Canada	June - October	*Microcystis*	[19]
Summer	Lake Steilacoom	America	July - September	*Microcystis aeruginosa*	[20]
Summer	Han River	Korea	August - September	*Anabaena, Microcystis*	[21]
Autumn	Han River	Korea	October	*Unruhdinium penardii*	[22]
Autumn	Lake Hulun	China	November	*Microcystis, Planktolyngbya*	[18]
Winter	North Pine Reservoir	Australia	-	*Aphanocapsa*	[23]
Winter	Lake Erie	America, Canada	-	*Diatom*	[24]
Winter	Lake Taihu	China	-	*Microcystis*	[25]
Winter	Lake Poyang	China		*Cyclotella*	[26]

Table S3. Comparison of algal detection methods for DWSS.

Category	Method	Mechanism	advantages	Main limitations	Reference
Morphology-based	Microscopy (light, fluorescence, AFM)	Direct visualization of cellular morphology; fluorescence staining for viability; AFM for nanoscale surface topography	Taxonomic resolution at genus/species level; live/dead cell distinction; nanoscale surface topography (AFM)	Time-consuming procedure; requirement of expert training; potential loss of colloidal cells during sample preparation	[37–40]
Cytochrome-based	Spectrophotometry	Quantifies extracted pigments by light absorbance following the Lambert-Beer law	Rapid measurement; quantitative analysis; low cost	Lack of taxonomic information; requirement of pigment extraction; inability to differentiate viable cells, non-viable cells, or cell aggregates	[37]
	HPLC	Separates and quantifies pigments by differential partitioning between mobile and stationary phases	High throughput; low detection limit	Lack of morphological and taxonomic information; requirement of pigment extraction	[37]
	In-situ fluorometry	Measures in-line fluorescence of chlorophyll or phycocyanin as a biomass proxy	Real-time monitoring; low measurement error after proper calibration	Susceptibility to environmental interference; requirement of calibration; lack of taxonomic resolution	[41]
Nucleic acid-based	qPCR	Amplifies species-specific DNA sequences to enable quantitative detection	High sensitivity; species-specific quantification; detection of low abundance targets	Requirement of species-specific primers; inability to assess viability; unknown DNA extraction efficiency for colloidal cells; laboratory-based operation	[37,42–45]
Other advanced	Remote sensing	Retrieves algal biomass and bloom extent from surface water reflectance spectra	Large spatial coverage; long-term monitoring; early warning of surface blooms	Cannot detect subsurface colloidal Inability to detect subsurface or colloidal algae; low spatial resolution for small water bodies	[46–48]
	Flow cytometry	Measures forward scatter (size), side scatter (granularity), and fluorescence of individual cells	Rapid, multi-parametric,	High instrument cost; lack of morphological information; unsuitability for in-situ monitoring	[49,50]

			and high-throughput analysis		
	Machine learning-based image analysis	Extracts morphological features from microscopic images to classify algae using trained neural networks	High classification accuracy after training; amenability to automation	Requirement of large annotated training dataset; performance sensitivity to algal growth stage; lack of training data for colloidal forms	[40,51,52]

Table S4. Common removal techniques for algae in drinking water sources.

Classification	Method	Mechanism	Advantages	Disadvantages	Reference
Physical method	Activated carbon adsorption	Numerous adsorption sites owing to huge specific surface area and complex pore structure	Low cost and can remove odorous substances simultaneously	Difficult to recover	[27]
	Mechanical salvage	Collection using power units	Rapid reduction	Difficulty in treating salvaged algae with higher water content	[28]
	Dredge	Removal of underwater silt containing nutrient	Alleviate water eutrophication	Difficulty in treating floating algae	[29]
	Hydrodynamic regulation	Accelerate mixing of water	Destroyed surface aggregation of algal cells	High energy consumption of pumps	[30]
	Ultrasounds	Destroy algal cells and gas vesicles	Rapid destruction of algae cells	High energy consumption and small effective distance	[31]
Chemical method	Coagulation	Neutralize surface charge and precipitate aggregates	Secure and easy to apply in the field	Large amounts of flocculant causing secondary pollution	[32]
	Chemical algaecide	Inhibited normal activities of algae	High efficiency	Secondary pollution	[33]
	Extracted allelochemicals	Affect cell structure and inhibit physiological processes	Negligible toxicity, high selectivity, and excellent degradability	Difficulty in extracting from plants and microorganisms	[30]
Biological method	Aquatic plants	Release allelochemicals, or compete nutrients and light	No chemical pollution and environmentally friendly	Long growth cycle	[34]
	Animal	Influence community structure through ingestion of zooplankton/filter-feeding fish	Non-toxic and environmentally friendly	Disrupt the original ecological balance	[35]
	Algaecidal bacteria	Disrupt physiology and metabolism by releasing bactericidal substances	Specificity	Cannot remove all algal species	[36]

Table S5. Comparison of algae removal technologies within DWTPs.

Process stage	Technology	Influencing factors	advantages	Disadvantages	Reference
Pre-treatment	Pre-oxidation (Cl_2, O_3, $KMnO_4$)	Oxidant type; dose; contact time; pH; temperature	Low-dose enhancement of coagulation; reduction of cell surface charge	Cell lysis and toxin release upon overdose; formation of disinfection by-products (DBPs)	[53,54]
	Dissolved Air Flotation (DAF)	Bubble size; coagulant type and dose; collision/attachment efficiency; hydraulic loading; floc size and density	High removal efficiency for low-density algae; large treatment capacity	Reduced effectiveness for small cells (< 5 μm); variability in bubble-cell attachment	[55]
	Ultrasonic treatment	Ultrasonic frequency; power density; exposure time	Rapid cell disruption	High energy consumption; cell lysis and toxin release due to shear forces; lab-scale studies only; infeasibility for DWTP scale	[56,57]
	Centrifugation	Centrifugal force; spin time	Rapid cell separation	High energy demand; cell lysis and toxin release; lab-scale only; infeasibility for DWTP scale	
Coagulation	Conventional coagulation (Al/Fe salts)	Coagulant type, dosage, and application scheme; mixing intensity; sedimentation time; pH, temperature, ionic strength, NOM; algal species, size, surface charge, EPS	Wide applicability; low cost; mature technology	Ineffectiveness for stable, negatively charged colloidal cells; high coagulant demand	[58,59]
	Enhanced coagulation (magnetic flocculants, novel coagulants)	Coagulant type and dosage; magnetic field strength (if applicable); water quality parameters	Improved removal of negatively charged colloidal cells; reduced coagulant demand (for some novel coagulants)	Higher material cost; potential residual magnetic particles; case-specific optimization	[58,60,61]
Sedimentation	Gravity sedimentation	Algal properties; hydraulic retention time	Simple operation; no energy input	Very slow settling for low-density or charged cells; ineffectiveness for colloidal algae	[2,62,63]
Filtration	Conventional media filtration	Filter media type, characteristics, and size; filtration velocity; bed depth	No chemical addition; simple operation	Rapid breakthrough and clogging; negligible	[59,64]

				removal of colloidal cells (cells pass through)	
Disinfection	Chemical disinfection (Cl_2, UV, O_3)	Disinfectant type, dose, and contact time; water quality (pH, temperature, ionic strength, coexisting substances); algal species	Final microbial barrier; residual disinfection capacity (Cl_2)	Not designed for bulk algal removal; chemical disinfectants cause cell lysis, toxin release, and DBP formation	[59,59,65]
After-treatment	Membrane filtration (UF/MF)	Membrane pore size ; transmembrane pressure; flux, fouling/cleaning (backwash); feed water quality	High removal efficiency via size exclusion; no cell lysis, resulting in low toxin release	Membrane fouling; high cost; concentrate disposal; unsuitability for high-turbidity feed water	[39,66]
	Advanced Oxidation Processes (AOPs) (e.g., electrochemical oxidation, photocatalysis, Fenton, activated persulfate, ozone-based AOPs)	Oxidant type and dosage; UV fluence; pH; reaction time; water quality; radical type and generation pathway; temperature; catalyst	Degradation of algal toxins and refractory organic compounds	High energy/chemical consumption; potential formation of toxic by-products; complex optimization	[59,67–69]

References:

1. Karthik, R.; Robin, R.S.; Anandavelu, I.; Purvaja, R.; Singh, G.; Mugilarasan, M.; Jayalakshmi, T.; Deepak Samuel, V.; Ramesh, R. Diatom bloom in the amba river, west coast of India: A nutrient-enriched tropical river-fed estuary. Regional Studies in Marine Science 2020, 35, 101244, doi:10.1016/j.rsma.2020.101244.
2. Zepernick, B.N.; Hart, L.N.; Chase, E.E.; Natwora, K.E.; Obuya, J.A.; Olokotum, M.; Houghton, K.A.; Kiledal, E.A.; Achieng, D.; Barker, K.B.; et al. Molecular investigation of harmful cyanobacteria reveals hidden risks and niche partitioning in kenyan lakes. Harmful Algae 2024, 140, 102757, doi:10.1016/j.hal.2024.102757.
3. Sitoki, L.; Kurmayer, R.; Rott, E. Spatial variation of phytoplankton composition, biovolume, and resulting microcystin concentrations in the nyanza gulf (lake victoria, kenya). Hydrobiologia 2012, 691, 109–122, doi:10.1007/s10750-012-1062-8.
4. Amorim, C.A.; Moura, A.D.N. Ecological impacts of freshwater algal blooms on water quality, plankton biodiversity, structure, and ecosystem functioning. Science of The Total Environment 2021, 758, 143605, doi:10.1016/j.scitotenv.2020.143605.
5. Mohamed, Z.A.; Mostafa, Y.; Alamri, S.; Hashem, M.; Alrumman, S. Ichthyotoxicity of bloom-forming dinoflagellates in an egyptian saline lake: Potential role of PUFA. Ecohydrology & Hydrobiology 2023, 23, 261–271, doi:10.1016/j.ecohyd.2022.11.003.
6. Sidibe, G.; Gan, L.; Liu, H.; Sumana, S.L.; Kamara, A.M.; Xu, L. Seasonal dynamics of phytoplankton communities in relation to water quality in poyang lake, China. Environments 2025, 12, 388, doi:10.3390/environments12100388.
7. Yang, J.; Gao, H.; Glibert, P.M.; Wang, Y.; Tong, M. Rates of nitrogen uptake by cyanobacterially-dominated assemblages in lake taihu, China, during late summer. Harmful Algae 2017, 65, 71–84, doi:10.1016/j.hal.2017.04.001.
8. Ji, D.; Han, Y.; Long, L.; Xin, X.; Xu, H.; Qiu, S.; Meng, J.; Zhao, X.; Huang, Y.; Liu, D. Hypoxia and its feedback response to algal blooms and CH4 emissions in subtropical reservoirs. Front. Ecol. Evol. 2024, 11, 1297047, doi:10.3389/fevo.2023.1297047.
9. Zhang, R.; Qi, F.; Liu, C.; Zhang, Y.; Wang, Y.; Song, Z.; Kumirska, J.; Sun, D. Cyanobacteria derived taste and odor characteristics in various lakes in China: Songhua lake, chaohu lake and taihu lake. Ecotoxicology and Environmental Safety 2019, 181, 499–507, doi:10.1016/j.ecoenv.2019.06.046.
10. Yancey, C.E.; Mathiesen, O.; Dick, G.J. Transcriptionally active nitrogen fixation and biosynthesis of diverse secondary metabolites by dolichospermum and aphanizomenon-like cyanobacteria in western lake erie microcystis blooms. Harmful Algae 2023, 124, 102408, doi:10.1016/j.hal.2023.102408.
11. O'Farrell, I.; Motta, C.; Forastier, M.; Polla, W.; Otaño, S.; Meichtry, N.; Devercelli, M.; Lombardo, R. Ecological meta-analysis of bloom-forming planktonic cyanobacteria in argentina. Harmful Algae 2019, 83, 1–13, doi:10.1016/j.hal.2019.01.004.
12. Cembella, A.; Klemm, K.; John, U.; Karlson, B.; Arneborg, L.; Clarke, D.; Yamanaka, T.; Cusack, C.; Naustvoll, L.; Bresnan, E.; et al. Emerging phylogeographic perspective on the toxigenic diatom genus

pseudo-nitzschia in coastal northern european waters and gateways to eastern arctic seas: Causes, ecological consequences and socio-economic impacts. Harmful Algae 2023, 129, 102496, doi:10.1016/j.hal.2023.102496.
13. Zhang, X.; Cvetkovska, M.; Morgan-Kiss, R.; Hüner, N.P.A.; Smith, D.R. Draft genome sequence of the antarctic green alga chlamydomonas sp. UWO241. iScience 2021, 24, 102084, doi:10.1016/j.isci.2021.102084.
14. Fang, X.; Yang, Z.; Ji, D.; Yao, X.; Liu, D. Responses of spring phytoplankton communities to their habitats in the xiangxi bay of three gorges reservoir, China. Acta Ecologica Sinica 2013, 33, 308–316, doi:10.1016/j.chnaes.2013.09.002.
15. Huang, T.; Wen, C.; Wang, S.; Wen, G.; Li, K.; Zhang, H.; Wang, Z. Controlling spring dinoflagellate blooms in a stratified drinking water reservoir via artificial mixing: Effects, mechanisms, and operational thresholds. Science of The Total Environment 2022, 847, 157400, doi:10.1016/j.scitotenv.2022.157400.
16. Jöhnk, K.D.; Huisman, J.; Sharples, J.; Sommeijer, B.; Visser, P.M.; Stroom, J.M. Summer heatwaves promote blooms of harmful cyanobacteria. Global Change Biology 2008, 14, 495–512, doi:10.1111/j.1365-2486.2007.01510.x.
17. Paerl, H.W.; Xu, H.; Hall, N.S.; Zhu, G.; Qin, B.; Wu, Y.; Rossignol, K.L.; Dong, L.; McCarthy, M.J.; Joyner, A.R. Controlling cyanobacterial blooms in hypertrophic lake taihu, China: Will nitrogen reductions cause replacement of non-N2 fixing by N2 fixing taxa? PLoS ONE 2014, 9, e113123, doi:10.1371/journal.pone.0113123.
18. Li, X.; Liu, Y.; Zhang, S.; Li, G.; Tao, Y.; Wang, S.; Yu, H.; Shi, X.; Zhao, S. Evolution characteristics and driving factors of cyanobacterial blooms in hulun lake from 2018 to 2022. Water 2023, 15, 3765, doi:10.3390/w15213765.
19. Wynne, T.; Stumpf, R. Spatial and temporal patterns in the seasonal distribution of toxic cyanobacteria in western lake erie from 2002–2014. Toxins 2015, 7, 1649–1663, doi:10.3390/toxins7051649.
20. Jacoby, J.M.; Collier, D.C.; Welch, E.B.; Hardy, F.J.; Crayton, M. Environmental factors associated with a toxic bloom of microcystis aeruginosa. Can. J. Fish. Aquat. Sci. 2000, 57, 231–240, doi:10.1139/f99-234.
21. Kim, J.; Lee, T.; Seo, D. Algal bloom prediction of the lower han river, korea using the EFDC hydrodynamic and water quality model. Ecological Modelling 2017, 366, 27–36, doi:10.1016/j.ecolmodel.2017.10.015.
22. Kim, T.; Ki, J.-S. Autumn blooms and seasonality of the dinoflagellate unruhdinium penardii in the han river (korea) as tracked by morphological and molecular techniques. Journal of Plankton Research 2022, 44, 194–207, doi:10.1093/plankt/fbac009.
23. Burford, M.A.; Mcneale, K.L.; Mckenzie-Smith, F.J. The role of nitrogen in promoting the toxic cyanophyte cylindrospermopsis raciborskii in a subtropical water reservoir. Freshwater Biology 2006, 51, 2143–2153, doi:10.1111/j.1365-2427.2006.01630.x.
24. Reavie, E.D.; Cai, M.; Twiss, M.R.; Carrick, H.J.; Davis, T.W.; Johengen, T.H.; Gossiaux, D.; Smith, D.E.; Palladino, D.; Burtner, A.; et al. Winter–spring diatom production in lake erie is an important driver of summer hypoxia. Journal of Great Lakes Research 2016, 42, 608–618, doi:10.1016/j.jglr.2016.02.013.
25. Ma, J.; Qin, B.; Paerl, H.W.; Brookes, J.D.; Hall, N.S.; Shi, K.; Zhou, Y.; Guo, J.; Li, Z.; Xu, H.; et al. The persistence of cyanobacterial (M icrocystis spp.) blooms throughout winter in lake taihu, China:

Cyanobacterial blooms throughout winter. Limnol. Oceanogr. 2016, 61, 711–722, doi:10.1002/lno.10246.
26. Li, B.; Li, J.; Hu, Y.; Cheng, S.; Li, S.; Zhang, X. Algal community dynamics in three water intakes of poyang lake: Implications for drinking water safety and management strategies. Water 2025, 17, 2034, doi:10.3390/w17132034.
27. Sichrova, K.; Cermakova, L.; Novotna, K.; Pivokonska, L.; Zustakova, V.; Pivokonsky, M. Mixed adsorbents: Synergic effects improve problematic compounds removal during drinking water treatment. AWWA Water Science 2025, 7, e70011, doi:10.1002/aws2.70011.
28. Zeng, G.; Zhang, R.; Liang, D.; Wang, F.; Han, Y.; Luo, Y.; Gao, P.; Wang, Q.; Wang, Q.; Yu, C.; et al. Comparison of the advantages and disadvantages of algae removal technology and its development status. Water 2023, 15, 1104, doi:10.3390/w15061104.
29. Wan, W.; Zhang, Y.; Cheng, G.; Li, X.; Qin, Y.; He, D. Dredging mitigates cyanobacterial bloom in eutrophic lake nanhu: Shifts in associations between the bacterioplankton community and sediment biogeochemistry. Environmental Research 2020, 188, 109799, doi:10.1016/j.envres.2020.109799.
30. Zhu, X.; Dao, G.; Tao, Y.; Zhan, X.; Hu, H. A review on control of harmful algal blooms by plant-derived allelochemicals. Journal of Hazardous Materials 2021, 401, 123403, doi:10.1016/j.jhazmat.2020.123403.
31. Lürling, M.; Tolman, Y. Beating the blues: Is there any music in fighting cyanobacteria with ultrasound? Water Research 2014, 66, 361–373, doi:10.1016/j.watres.2014.08.043.
32. Naceradska, J.; Semerad, J.; Cajthaml, T.; Pivokonsky, M. Removal of natural bloom algal organic matter and associated toxins by coagulation and ozonation. Ozone: Science & Engineering 2025, 1–14, doi:10.1080/01919512.2025.2586184.
33. Lad, A.; Breidenbach, J.D.; Su, R.C.; Murray, J.; Kuang, R.; Mascarenhas, A.; Najjar, J.; Patel, S.; Hegde, P.; Youssef, M.; et al. As we drink and breathe: Adverse health effects of microcystins and other harmful algal bloom toxins in the liver, gut, lungs and beyond. Life 2022, 12, 418, doi:10.3390/life12030418.
34. Wang, B.; Zhang, Y.; Qin, Y.; Li, H. Removal of microcystis aeruginosa and control of algal organic matter by fe(II)/peroxymonosulfate pre-oxidation enhanced coagulation. Chemical Engineering Journal 2021, 403, 126381, doi:10.1016/j.cej.2020.126381.
35. Li, J.; Gu, H.; Lovko, V.J.; Liang, C.; Li, X.; Xu, X.; Jia, L.; Jiang, M.; Wang, J.; Chen, J. The ciliate euplotes balteatus exhibits removal capacity upon the dinoflagellates karenia mikimotoi and prorocentrum shikokuense. Harmful Algae 2024, 138, 102685, doi:10.1016/j.hal.2024.102685.
36. Yu, Y.; Zeng, Y.; Li, J.; Yang, C.; Zhang, X.; Luo, F.; Dai, X. An algicidal streptomyces amritsarensis strain against microcystis aeruginosa strongly inhibits microcystin synthesis simultaneously. Science of The Total Environment 2019, 650, 34–43, doi:10.1016/j.scitotenv.2018.08.433.
37. Liu, F.; Zhang, C.; Wang, Y.; Chen, G. A review of the current and emerging detection methods of marine harmful microalgae. Science of The Total Environment 2022, 815, 152913, doi:10.1016/j.scitotenv.2022.152913.
38. Fiołka, M.J.; Sofińska-Chmiel, W.; Procházková, L.; Mieszawska, S.; Dryglewska, M.; Skrzypiec, K.; Wydrych, J. Morphological and environmental analysis of the glacier ice alga ancylonema alaskanum. Sci Rep 2025, 15, 18578, doi:10.1038/s41598-025-95754-9.

39. Villacorte, L.O.; Ekowati, Y.; Neu, T.R.; Kleijn, J.M.; Winters, H.; Amy, G.; Schippers, J.C.; Kennedy, M.D. Characterisation of algal organic matter produced by bloom-forming marine and freshwater algae. Water Research 2015, 73, 216–230, doi:10.1016/j.watres.2015.01.028.
40. Chong, J.W.R.; Khoo, K.S.; Chew, K.W.; Vo, D.-V.N.; Balakrishnan, D.; Banat, F.; Munawaroh, H.S.H.; Iwamoto, K.; Show, P.L. Microalgae identification: Future of image processing and digital algorithm. Bioresource Technology 2023, 369, 128418, doi:10.1016/j.biortech.2022.128418.
41. Choo, F.; Zamyadi, A.; Stuetz, R.M.; Newcombe, G.; Newton, K.; Henderson, R.K. Enhanced real-time cyanobacterial fluorescence monitoring through chlorophyll-a interference compensation corrections. Water Research 2019, 148, 86–96, doi:10.1016/j.watres.2018.10.034.
42. Aguilera, A.; Almanza, V.; Haakonsson, S.; Palacio, H.; Benitez Rodas, G.A.; Barros, M.U.G.; Capelo-Neto, J.; Urrutia, R.; Aubriot, L.; Bonilla, S. Cyanobacterial bloom monitoring and assessment in latin America. Harmful Algae 2023, 125, 102429, doi:10.1016/j.hal.2023.102429.
43. Durán-Vinet, B.; Araya-Castro, K.; Chao, T.; Wood, S.; Gallardo, V.; Godoy, K.; Abanto, M. Potential applications of CRISPR/cas for next-generation biomonitoring of harmful algae blooms: A review. Harmful Algae 2021, 103, 102027, doi:10.1016/j.hal.2021.102027.
44. Bailet, B.; Apothéloz-Perret-Gentil, L.; Baričević, A.; Chonova, T.; Franc, A.; Frigerio, J.-M.; Kelly, M.; Mora, D.; Pfannkuchen, M.; Proft, S.; et al. Diatom DNA metabarcoding for ecological assessment: Comparison among bioinformatics pipelines used in six european countries reveals the need for standardization. Science of The Total Environment 2020, 745, 140948, doi:10.1016/j.scitotenv.2020.140948.
45. Medlin, L.; Orozco, J. Molecular techniques for the detection of organisms in aquatic environments, with emphasis on harmful algal bloom species. Sensors 2017, 17, 1184, doi:10.3390/s17051184.
46. Wang, W.; Shi, K.; Zhang, Y.; Li, N.; Sun, X.; Zhang, D.; Zhang, Y.; Qin, B.; Zhu, G. A ground-based remote sensing system for high-frequency and real-time monitoring of phytoplankton blooms. Journal of Hazardous Materials 2022, 439, 129623, doi:10.1016/j.jhazmat.2022.129623.
47. Li, D.; Yu, D.; Xu, Y.; Jia, P.; Xue, W. Automatic extraction method of green tide based on mixed pixel decomposition feedback adjustment. IEEE J. Sel. Top. Appl. Earth Observations Remote Sensing 2025, 18, 5975–5989, doi:10.1109/JSTARS.2024.3504561.
48. Dev, P.J.; Sukenik, A.; Mishra, D.R.; Ostrovsky, I. Cyanobacterial pigment concentrations in inland waters: Novel semi-analytical algorithms for multi- and hyperspectral remote sensing data. Science of The Total Environment 2022, 805, 150423, doi:10.1016/j.scitotenv.2021.150423.
49. Buskey, E.J.; Hyatt, C.J. Use of the FlowCAM for semi-automated recognition and enumeration of red tide cells (karenia brevis) in natural plankton samples. Harmful Algae 2006, 5, 685–692, doi:10.1016/j.hal.2006.02.003.
50. Latasa, M.; Scharek, R.; Morán, X.A.G.; Gutiérrez-Rodríguez, A.; Emelianov, M.; Salat, J.; Vidal, M.; Estrada, M. Dynamics of phytoplankton groups in three contrasting situations of the open NW mediterranean sea revealed by pigment, microscopy, and flow cytometry analyses. Progress in Oceanography 2022, 201, 102737, doi:10.1016/j.pocean.2021.102737.
51. Ning, H.; Li, R.; Zhou, T. Machine learning for microalgae detection and utilization. Front. Mar. Sci.

2022, 9, 947394, doi:10.3389/fmars.2022.947394.
52. Yadav, D.P.; Jalal, A.S.; Garlapati, D.; Hossain, K.; Goyal, A.; Pant, G. Deep learning-based ResNeXt model in phycological studies for future. Algal Research 2020, 50, 102018, doi:10.1016/j.algal.2020.102018.
53. Xie, P.; Chen, Y.; Ma, J.; Zhang, X.; Zou, J.; Wang, Z. A mini review of preoxidation to improve coagulation. Chemosphere 2016, 155, 550–563, doi:10.1016/j.chemosphere.2016.04.003.
54. Ma, W.; Li, X.; Li, D.; Wang, L.; Li, S.; Li, J.; Ma, W.; Li, Z.; Ding, C. Algal-rich drinking source water: Effects of chlorine pre-oxidation on algal growth, algal organic matter, and the potential of disinfection by-products. Water Air Soil Pollut 2024, 235, 239, doi:10.1007/s11270-024-07041-4.
55. Qi, J.; Lan, H.; Liu, R.; Liu, H.; Qu, J. Fe(II)-regulated moderate pre-oxidation of microcystis aeruginosa and formation of size-controlled algae flocs for efficient flotation of algae cell and organic matter. Water Research 2018, 137, 57–63, doi:10.1016/j.watres.2018.03.005.
56. Liu, J.-Y.; Zeng, L.-H.; Ren, Z.-H.; Du, T.-M.; Liu, X. Rapid in situ measurements of algal cell concentrations using an artificial neural network and single-excitation fluorescence spectrometry. Algal Research 2020, 45, 101739, doi:10.1016/j.algal.2019.101739.
57. Peng, Y.; Xiao, X.; Ren, B.; Zhang, Z.; Shi, X.; Wang, C.; Zhang, W. Removal of anabaena by ultrasonic pretreatment enhancing-coagulation and water treatment processes. Journal of Cleaner Production 2024, 447, 141521, doi:10.1016/j.jclepro.2024.141521.
58. Ma, J.; Xia, W.; Fu, X.; Ding, L.; Kong, Y.; Zhang, H.; Fu, K. Magnetic flocculation of algae-laden raw water and removal of extracellular organic matter by using composite flocculant of Fe3O4/cationic polyacrylamide. Journal of Cleaner Production 2020, 248, 119276, doi:10.1016/j.jclepro.2019.119276.
59. He, X.; Liu, Y.-L.; Conklin, A.; Westrick, J.; Weavers, L.K.; Dionysiou, D.D.; Lenhart, J.J.; Mouser, P.J.; Szlag, D.; Walker, H.W. Toxic cyanobacteria and drinking water: Impacts, detection, and treatment. Harmful Algae 2016, 54, 174–193, doi:10.1016/j.hal.2016.01.001.
60. Bian, Y.; Li, S.; Luo, H.; Lv, L.; Zan, S.; Ren, B.; Zhu, G. Magnetic metal–organic framework enhanced inorganic coagulation for water purification. Water 2023, 15, 3391, doi:10.3390/w15193391.
61. Li, Q.; Zhong, Y.; Zhang, Y.; Liu, W.; Tian, Y.; Qin, T.; Wu, M.; Xiong, W.; Chen, Z.; Zhou, Y.; et al. Simple synthesis and excellent coagulation performance of a novel red soil-based coagulant. Separation and Purification Technology 2025, 359, 130846, doi:10.1016/j.seppur.2024.130846.
62. Ge, S.; Agbakpe, M.; Wu, Z.; Kuang, L.; Zhang, W.; Wang, X. Influences of surface coating, UV irradiation and magnetic field on the algae removal using magnetite nanoparticles. Environ. Sci. Technol. 2015, 49, 1190–1196, doi:10.1021/es5049573.
63. Zhao, W.; Zhao, P.; Tian, Y.; Shen, C.; Li, Z.; Peng, P.; Jin, C. Investigation for synergies of ionic strength and flow velocity on colloidal-sized microplastic transport and deposition in porous media using the colloidal–AFM probe. Langmuir 2020, 36, 6292–6303, doi:10.1021/acs.langmuir.0c00116.
64. Zhao, W.; Zhao, P.; Tian, Y.; Shen, C.; Li, Z.; Jin, C. Transport and retention of microcystis aeruginosa in porous media: Impacts of ionic strength, flow rate, media size and pre-oxidization. Water Research 2019, 162, 277–287, doi:10.1016/j.watres.2019.07.001.
65. Huang, R.; Liu, Z.; Yan, B.; Zhang, J.; Liu, D.; Xu, Y.; Wang, P.; Cui, F.; Liu, Z. Formation kinetics of

disinfection byproducts in algal-laden water during chlorination: A new insight into evaluating disinfection formation risk. Environmental Pollution 2019, 245, 63–70, doi:10.1016/j.envpol.2018.10.074.
66. Kong, X.; Ma, J.; Le-Clech, P.; Wang, Z.; Tang, C.Y.; Waite, T.D. Management of concentrate and waste streams for membrane-based algal separation in water treatment: A review. Water Research 2020, 183, 115969, doi:10.1016/j.watres.2020.115969.
67. Lu, S.; Zhang, G. Recent advances on inactivation of waterborne pathogenic microorganisms by (photo) electrochemical oxidation processes: Design and application strategies. Journal of Hazardous Materials 2022, 431, 128619, doi:10.1016/j.jhazmat.2022.128619.
68. Zhang, H.; Yu, B.; Li, X.; Li, Y.; Zhong, Y.; Ding, J. Inactivation of microcystis aeruginosa by peroxydisulfate activated with single-atomic iron catalysis: Efficiency and mechanisms. Journal of Environmental Chemical Engineering 2022, 10, 108310, doi:10.1016/j.jece.2022.108310.
69. Li, Z.; Chen, J.; Wang, C.; Zhao, J.; Wei, Q.; Ma, X.; Yang, G. Study on the removal and degradation mechanism of microcystin-LR by the UV/fenton system. Science of The Total Environment 2023, 892, 164665, doi:10.1016/j.scitotenv.2023.164665.
